# The Harmful Effects of Subarachnoid Hemorrhage on Extracerebral Organs

**DOI:** 10.1155/2014/858496

**Published:** 2014-07-07

**Authors:** Sheng Chen, Qian Li, Haijian Wu, Paul R. Krafft, Zhen Wang, John H. Zhang

**Affiliations:** ^1^Department of Neurosurgery, Second Affiliated Hospital, School of Medicine, Zhejiang University, Hangzhou, Zhejiang 310009, China; ^2^Department of Physiology and Pharmacology, Loma Linda University School of Medicine, Loma Linda, CA 92354, USA; ^3^Department of Neurology, The Fifth People's Hospital, Chongqing 400011, China

## Abstract

Subarachnoid hemorrhage (SAH) is a devastating neurological disorder. Patients with aneurysmal SAH develop secondary complications that are important causes of morbidity and mortality. Aside from secondary neurological injuries, SAH has been associated with nonneurologic medical complications, such as neurocardiogenic injury, neurogenic pulmonary edema, hyperglycemia, and electrolyte imbalance, of which cardiac and pulmonary complications are most common. The related mechanisms include activation of the sympathetic nervous system, release of catecholamines and other hormones, and inflammatory responses. Extracerebral complications are directly related to the severity of SAH-induced brain injury and indicate the clinical outcome in patients. This review provides an overview of the extracerebral complications after SAH. We also aim to describe the manifestations, underlying mechanisms, and the effects of those extracerebral complications on outcome following SAH.

## 1. Introduction

The prevalence of unruptured intracranial aneurysms in health adults was found to be between 3% and 7% [1, 2]. Spontaneous rupture of intracranial aneurysms may lead to subarachnoid hemorrhage (SAH), a hemorrhagic stroke subtype with a high case fatality [1]. Nearly 30,000 individuals in the United States are affected by aneurysmal SAH each year [3]. Although early surgical or endovascular repair of ruptured aneurysms and aggressive postoperative management has improved the overall outcome in patients, SAH continues to be responsible for physical, psychological, and financial damage in developing and developed countries alike. Thus, SAH remains a worldwide leading cause of death and neurological disability. Indeed, the mortality rate is approaching 50% and less than 60% of SAH survivors return to functional independence [4, 5]. Neurologic outcome following SAH is largely determined by the amount and location of initial bleeding. Previous studies have focused on intracranial complications of SAH as independent predictors of outcome, such as early brain injury, delayed cerebral ischemia, and chronic hydrocephalus [5–9].

**Figure 1:**
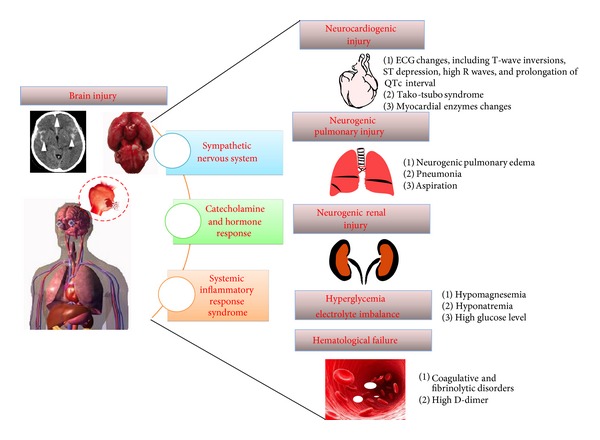
Schematic of nonneurologic medical complications following subarachnoid hemorrhage.

Aside from the primary and secondary neurological injury induced by this stroke subtype, SAH is also significantly associated with nonneurologic medical complications. Indeed, SAH patients are extremely vulnerable to multiple extracerebral organ dysfunctions (Figure 1). With improvements in the surgical and endovascular management of intracranial aneurysms, nonneurological complications will assume a more prominent role in the overall outcome of SAH patients [10], as such complications may increase the length of hospital stays as well as the need of intensive care unit management. Evidently, nonneurological organ dysfunctions correlate with the severity of brain injury following SAH. The most frequent nonneurologic medical complications occurring after SAH include pulmonary edema and pneumonia, cardiac arrhythmia, renal and hepatic dysfunction, electrolyte disturbance, and hematologic derangements [10]. Combinations of brain injury and extracerebral organ dysfunction may occur concurrently after SAH, and the latter may exacerbate brain injury during the acute phase of bleeding. Therefore, the prevention and management of nonneurological complications are important for improving the overall clinical outcome after SAH.

This review focuses on nonneurologic complications after SAH, describing the frequency, severity, and manifestations of those complications. In particular, we discussed several underlying mechanisms of the nonneurologic complications and present treatment opportunities.

## 2. Possible Mechanisms of Nonneurologic Complications following SAH

### 2.1. Hormone Response to SAH

Psychological and physical insults to the central nervous system can trigger a disastrous response of the sympathetic nervous system, eventually leading to end-organ catecholamine-mediated injury [11, 12]. Massive sympathetic nervous activation occurs in SAH patients. Activation of the sympathetic nervous system, which leads to an elevated level of circulating, cerebrospinal fluid (CSF), and urine catecholamines, may be the link between the initial ictus and the genesis of some of the systemic complications after SAH [13]. Sympathetic activation was observed as an elevation of plasma norepinephrine following preclinical and clinical SAH studies [14, 15]. It has been well recognized that a high sympathetic tone combined with high circulating catecholamine concentrations may occur in humans with head injury, particularly after SAH [13, 16]. The amount of catecholamines released into the systemic circulation of SAH patients was found even higher than in patients with cardiac arrest or asphyxia [17]. Meanwhile, the uptake of norepinephrine was found to be decreased following SAH. Physiological derangements can occur following a sudden and sustained increase in systemic catecholamines. Catecholamines potentiate the activation of endothelin [18], which plays a role in the development of vasospasms. It has been reported that endothelin-induced cerebral vasospasms were associated with delayed cerebral ischemia following SAH [19, 20]. However, the randomized, double-blinded, placebo-controlled, phase 3 study (CONSCIOUS-2 and CONSCIOUS-3) demonstrated that the endothelin-1 receptor antagonist clazosentan decreased the occurrence of cerebral vasospasm but had no significant effect on the functional outcome after SAH [21, 22]. Catecholamine-induced stress may be associated with the well-known organ dysfunction described in SAH with the production of toxic cytokines, including high-pressure pulmonary edema, myocardial myocytolysis, stress hyperglycemia, hypokalemia, and leukocytosis (10753996, 1604280). Data from animal models and clinical studies suggest that the increased release in catecholamines is the most likely underlying cause of cardiac injury after SAH [15, 23]. The “catecholamine hypothesis” is particularly supported by an experimental model of sudden brain death, which demonstrated immediate and massive increases in myocardial norepinephrine measured by microdialysis techniques [24]. Furthermore, hormonal profiles of SAH patients demonstrated an increase in natriuretic peptide, renin, angiotensin II accompanied with high concentration of cardiac troponin I (cTnI), and stable low levels of vasopressin. Both brain natriuretic peptide (BNP) and atrial natriuretic peptide (ANP) levels in SAH patients were found to be elevated to values 2-3 times greater than those observed in healthy volunteers within 3-4 days after the ictus [25]. However, in another study, throughout the 7 days after SAH, lower than normal aldosterone concentrations and normal plasma concentrations of ANP and C-type natriuretic peptides (CNP) were found [26]. B-type natriuretic peptide showed significant diagnostic efficiency for predicting delayed cerebral ischemia after SAH [27]. Rapid natriuresis occurs prior to the development of ischemic symptoms after SAH, indicating that it is a trigger for symptomatic vasospasm [28]. Cerebral salt wasting concomitantly occurs following SAH, which induces excessive natriuresis and osmotic diuresis. Natriuresis results in the reduction of total blood volume and increases the risk of symptomatic vasospasm after SAH in patients as well as in an SAH rodent model [29, 30]. Increased levels of both ANP and BNP were found in SAH patients, which surprisingly were not related to either biomarkers or clinical severity of cardiac injury [25]. The levels of plasma natriuretic peptides were much higher than CSF levels of natriuretic peptides, which supported the view that the heart is the source of plasma ANP and BNP after SAH [25].

An approximately 3-fold increase in plasma renin activity was observed by measuring the level of angiotensin I, which indicates an acute activation of the renin-angiotensin system in the early stages following experimental SAH [31]. Significant correlation was found between urinary catecholamines excretion and both plasma renin and plasma angiotensin II concentrations. SAH patients presenting elevated plasma renin levels experienced a higher incidence of mortality and morbidity than those with lower plasma renin [32], which indicates that the renin-angiotensin system may play a role in some of the deleterious consequences of SAH [33, 34]. Angiotensin II is of importance in disruption of the blood-brain barrier and the regulation of brain capillaries permeability following SAH [35, 36]; thus it may be a link between brain injury and extracerebral organs injury. Moreover, in studies of experimental SAH, delayed cerebral vasospasm was attenuated by the treatment of an angiotensin-converting enzyme inhibitor [37]. Angiotensin receptor blockade via losartan markedly decreased the survival in experimental SAH study, suggesting that the acute activation of the renin-angiotensin system is a desirably compensatory response [31].

Taken together, hormonal changes are implicated in the pathophysiology of SAH and their influence in the pathogenesis of delayed extracerebral complications warrants further investigation.

### 2.2. Systemic Inflammatory Response Syndrome (SIRS)

Inflammatory responses and metabolic derangements are frequently described in SAH patients [38]. These patients will often present febrile and tachycardic without underlying infections [39]. Systemic inflammatory response syndrome (SIRS) is an inflammatory phenomenon affecting the whole body, frequently a response of the immune system to infectious and noninfectious insults. SIRS accompanies various acute cerebral insults, including ischemic stroke, SAH, and intracerebral hemorrhage. SAH, as a noninfectious insult, can induce the SIRS via triggering immune system activation [40]. The surge in ICP and activation of the sympathetic nervous system contribute to SAH-induced SIRS [41]. Furthermore, patients undergoing aneurysm surgery have an increased likelihood of developing SIRS [41, 42]. Given the frequency of systemic disturbances, it has been reported that SIRS occurs with an incidence from 29% to 87% in SAH patients [43, 44]. In addition, SAH is frequently accompanied by leukocytosis, elevated levels of proinflammatory cytokines, and fever [45, 46]. Elevated levels of interleukin-6 (IL-6) and C-reactive protein (CRP) were found in the systemic circulation of SAH patients, with even higher peaks associated with delayed brain ischemia [47]. SIRS standard criteria include abnormal heart rate, respiration rate, temperature, and white blood cell count [48]. In a retrospective study, admission SIRS score proved to parallel the severity of SAH, indicated by Hunt and Hess grading, amount of clot demonstrated by radiographic examination, and plasma glucose concentration [40]. SIRS not only promoted extracerebral organ dysfunction, but also exacerbated delayed cerebral ischemia, contributing to a worsen outcome. Furthermore, SIRS contributed to acute lung injury and poor outcome after SAH [41, 49]. The admission SIRS score may be a significant outcome predictor of subsequent neurological deterioration.

## 3. Manifestations of Nonneurologic Complications after SAH

### 3.1. Neurocardiogenic Injury

Cardiac manifestations of SAH are particularly impressive, because manipulation of blood pressure parameters is routinely used as the treatment for SAH patients. In 1982, Braunwald and Kloner first defined the condition of “stunned myocardium,” as a reversible postischemic myocardial dysfunction [50]. Recently, neurocardiogenic injury following SAH has been further elucidated; it includes electrocardiographic (ECG) abnormalities, arrhythmias, myocardial infarction (both non-ST elevation and ST-elevation), left ventricular (LV) dysfunction, elevation of cTnI, and even cardiac arrest [51–54]. Those conditions have been considered in relation to SAH, although their clinical relevance is still unclear [51]. Significant cardiac dysfunction or laboratory evidence of cardiac injury complicates the management of SAH patients [55]. Moreover, pathological evidence of contraction band necrosis provided evidence for the development of myocardial necrosis in heart autopsies [56]. SAH patients with cardiac injury have higher short- and long-term mortality rates [57]. Several methods are used to identify myocardial injury, such as serial ECG, hemodynamic measurements, coronary angiography, blood flow measurements by radiolabeled microspheres, 2D echocardiography, and myocardial contrast echocardiography.

A variety of ECG changes, including T-wave inversions, ST depression, high R waves, prolongation of the corrected QT (QTc) interval, and large U waves, have been frequently documented in SAH patients, possibly because of elevated catecholamines or electrolyte imbalances. But the results of several studies demonstrated a negative relation between high levels of catecholamines and ECG changes [58, 59]. In addition, hypothalamic stimulation may induce ECG abnormalities without associated myocardial injury [60]. Furthermore, neurons of the nodose ganglia are damaged due to ischemic insult secondary to SAH. The ischemic neuronal degeneration in the nodose ganglia disturbed the afferent vagal nerve reflexes and eventually led to heart rhythm irregularities [61]. Evidence has accumulated and suggests that ECG abnormalities in the acute stage of SAH reflect a transient cardiac dysfunction rather than permanent myocardial injury. In a prospective study of 447 SAH patients, 39% of these patients experienced prolonged elevated heart rate (>95 beats/min for >12 h), which was associated with major adverse cardiopulmonary events and poor outcome after SAH [62]. Heart rate variability is a potential marker of reversible cardiac injury, severe vasospasm, and death [63–65]. In another study, 100 subjects who were admitted within 24 h after SAH demonstrated prolongation of the QTc interval. Further univariate analyses showed significant correlation between QTc interval length and other variables, such as sex, serum concentrations of potassium, calcium, or glucose. Nevertheless, these analyses suggested that only female sex and hypokalemia were an independent risk factor for severe QTc prolongation in SAH patients [66]. It has been confirmed that QTc interval prolongation improved in patients with a good prognosis; it persisted in SAH patients with a poor outcome, further indicating that a QTc interval of longer than 448 ms at 7 days after surgery can serve as a predictor of clinical outcome following SAH [67]. Twenty-three patients with SAH were examined, who showed an ST segment elevation in their ECG [68]. ECG and echocardiogram abnormalities were normalized and normalization of the apical wall motion was recorded on echocardiograms within several months after SAH, which indicated that cardiac dysfunction may be reversible. Previously, Kolin and Norris indicated that the distinctive myocardial lesion accompanying cerebral injury is reversible, because the increased level of catecholamines returned to the normal [69]. Early ECG abnormalities were associated with the in-hospital mortality of the patients with SAH, but not with the overall prognosis [70, 71].

Stress cardiomyopathy reflects merely a single aspect of a much wider range of neurocardiogenic injury, which encompasses cardiac dysfunction associated with SAH. Tako-tsubo syndrome is a rare acquired cardiomyopathy, characterized by LV dyskinesia and symptomatology typical of acute myocardial infarction. Although the pathogenesis of takot-subo syndrome has not yet been established, compelling literature supports the theory that acute cardiac sympathetic disruption accompanied with norepinephrine seethe and spillover are the mechanisms of tako-tsubo syndrome [72]. Historically, cardiac pathophysiology after SAH has been attributed to LV myocardial ischemia, which may be caused by coronary artery spasm and thrombosis and/or oxygen supply-demand mismatch in the setting of hypertension and tachycardia [73, 74]. Approximately 10% of all SAH patients suffer from LV systolic dysfunction [68]. A 54-year-old woman initially presented with ST elevation myocardial infarction and resultant LV failure, which was ultimately explained by the diagnosis of SAH with subsequent adrenergic storm [75]. Systolic dysfunction can be observed by echocardiography as a reduced LV ejection fraction and/or the presence of regional wall motion abnormalities of the LV. LV ejection fraction and pulse-wave velocity were related to poor outcomes following SAH [27]. A multicenter prospective cohort study found that the cardiac index was significantly lower in patients with high grade SAH (World Federation of Neurological Surgeons grades IV and V) on days 1 and 2 after the ictus [76]. This was further supported by a recent retrospective study, which highlighted the effect of norepinephrine in pathogenesis of SAH-induced wall motion abnormalities [15, 77]. In addition, postmenopausal women after poor-grade SAH are predisposed to develop wall motion abnormalities due to lack of estradiol [23]. Impaired LV hemodynamic performance was proposed to contribute to cardiovascular instability, pulmonary edema formation, and complications of cerebral ischemia [78].

Elevations in serum cardiac enzymes, including creatine kinase, MB isoenzyme (CK-MB), and cTnI, were elevated following SAH [79, 80]. Previous studies have shown that 17 to 28% of SAH patients develop elevated serum levels of cTnI [81, 82]. In severely affected patients with elevated levels of cTnI, reduction of cardiac output may increase the risk of cerebral ischemia and poor outcome related to vasospasm [81]. In a prospective study, cTnI has been shown to be a more sensitive indicator as compared to CK-MB in the detection of left ventricular dysfunction in patients with SAH [81]. A retrospective study including 617 consecutive SAH patients demonstrated that patients with high troponin levels demonstrated an increase in mortality [51]. In an SAH rat model, early activation of matrix metalloproteinases was observed in the myocardial tissue and plasma, which may enhance cTnI degradation [83]. Thus, matrix metalloproteinases antagonism may provide a protective effect against SAH-induced cardiac damage.

Interestingly, it has been reported that endovascular coiling or surgical clipping of ruptured aneurysms is not associated with the incidence of cardiac injury or dysfunction [84]. However, it is important to note that treatment decisions were made on the basis of standard practice patterns rather than on a randomization process. Intraoperative anesthetic management differs between the two procedures mentioned above. Additionally, patients who underwent aneurysm coiling were also treated with either anticoagulant or antiplatelet agents.

It is important to discover promising strategies that minimize neurocardiogenic complications. All patients with SAH require close cardiac monitoring, and, in some cases, cardiac *β*-adrenergic stimulation may be advisable.

### 3.2. Neurogenic Pulmonary Edema

**Figure 2:**
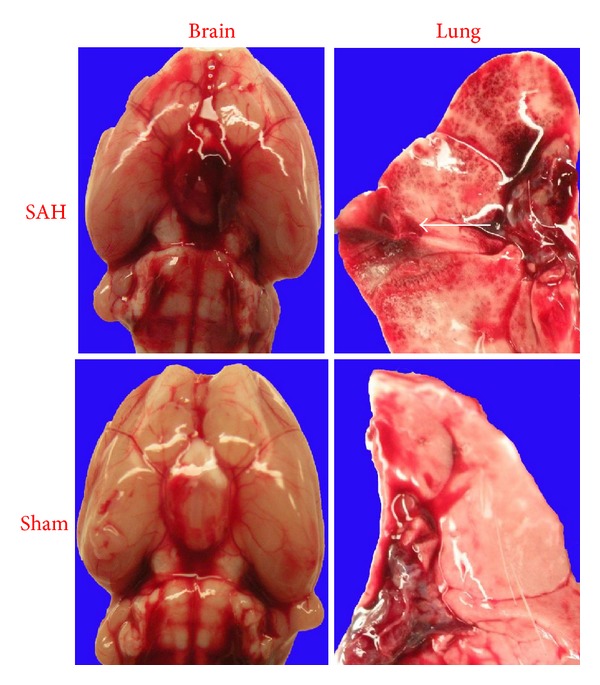
High-resolution pictures of subarachnoid hemorrhage and sporadic pulmonary hemorrhagic lesions in a rat endovascular puncture model (white arrow).

Pulmonary complications after SAH are a cluster of lung dysfunctions, which includes pneumonia, aspiration, and neurogenic pulmonary edema (NPE) (Figure 2) [85]. Pulmonary complications are the most frequent extracerebral cause of death after SAH [44, 86]. Oxygenation deficits occur in the acute stage of SAH. Indeed, oxygenation disturbances were found in 43% to 92% of SAH patients, which most often resulted from pulmonary edema. Patients with World Federation of Neurological Surgeons IV and V were significantly higher scored in the extravascular lung water index, pulmonary vascular permeability index, and systemic vascular resistance index on day 2 after SAH [76]. Differential diagnosis of the pulmonary complications can be difficult. NPE is usually suspected when there are no underlying lung diseases, and NPE is found in 23% to 71% patients during hospitalization [10, 87]. The incidence of pathological diagnosis of NPE is higher than its clinical diagnosis. The abnormality of NPE after SAH is often unilateral on chest X-ray. SAH patients with NPE were usually younger and died sooner than those without. The development of pulmonary edema most frequently occurs within the first week from the beginning of the SAH with a peak around day 3. The incidence of NPE decreased with time after SAH.

NPE displayed biphasic in SAH patients, first with cardiogenic NPE caused by cardiac dysfunction immediately after SAH, and hydrostatic NPE resulted from hypervolemia and low cardiac contractility 7 days after SAH [88]. NPE in SAH patients occurred for some mechanisms. First, at high pressure, disruption of the capillary endothelium and alveolar epithelium will occur due to raised capillary pressure with the development of a high-permeability of blood-lung barrier. A hydrostatic form of pulmonary edema develops. High-pressure pulmonary edema is apparently not the only mechanism. Secondly, a reversible form of cardiac injury is linked to NPE following SAH. Severe depression of left myocardial function occurring after SAH was regarded as another mechanism involved in NPE pathogenesis, as demonstrated in a retrospective study of 20 patients with NPE [89]. This is evident with most NPE patients demonstrating increased pulmonary wedge pressure and reduced cardiac output or reduced left ventricular function [78]. However, in a small sample-size retrospective study, there was no evidence for high-permeability edema or cardiac failure in half of patients who presented with oxygenation disturbances. In those patients, pulmonary edema may be due to extravascular lung water [90], because the latter was significantly and positively correlated with impaired oxygenation in a study of patients with hemorrhagic stroke [91]. Thirdly, some molecules, such as S100B, E-selectin, and caspase-1, can be the link between the brain and the lung that determine the development of NPE after SAH. S100B binds the receptor for advanced glycation end products in alveolar epithelial type I pneumocytes to amplify the immune and inflammatory response causing lung injury [92]. In an SAH mouse model, pulmonary endothelial cell apoptosis contributed to the pathophysiology of NPE [93]. Caspase-1 inhibitor can prevent the apoptosis of pulmonary endothelial cells and ameliorate NPE [94]. SAH increased the pulmonary expression of the cytokines (tumor necrosis factor-*α*), chemokines, and adhesion molecules (E-selectin, intercellular adhesion molecule- (ICAM-) 1, and vascular cell adhesion molecule- (VCAM-) 1). Interferon-*β* reduced lung inflammation following experimental SAH [95]. P2X purinoceptor 7 antagonist administration attenuates inflammation and prevents the lung-blood barrier in experimental SAH model [96]. Forth, the application of hypothermia and barbiturates in confronting high ICP may result in immune suppression, decreased leukocyte counts, and likely predisposes to pneumonia [97]. Additionally, diminished level of consciousness resulted in aspiration and impaired cough due to neurological injury. Sedation may also result in atelectasis. Furthermore, recently, vasospasm after SAH has been shown to lead to ischemic neurodegeneration in the dorsal root ganglia of the phrenic nerve, and phrenic nerve root ischemia has been suggested to play a crucial role in respiration rhythms deteriorations following experimental SAH [98]. Finally, overload of blood volume may be another contributing factor of pulmonary edema as this is generally the first intervention to maintain cerebral perfusion pressure or to ameliorate vasospasm due to aneurysmal bleeding. Recently, Mutoh et al. reported a new bedside transpulmonary thermodilution device, which is capable of distinguishing different etiologies and making fluid management decisions [99].

The majority of previous studies pay particular attention to pulmonary and cardiac dysfunction, but the burden of extracerebral organ failure after SAH, including renal, hematology, and liver, remains largely unstudied [44, 100].

### 3.3. Hyperglycemia and Electrolyte Imbalance

Stress hyperglycemia is present at admission in 70 to 90% of all SAH patients [101, 102]. For the mechanisms, the activation of the hepatic and pancreatic sympathetic nerve fibers resulted in increased output of glucose from the liver, a stimulation of glucagon, and an inhibition of insulin release from the pancreas [103]. Recent study suggested that catecholamine is involved in the development of hepatic insulin resistance via proinflammatory pathways [104].

Hyperglycemia exacerbates SAH-induced brain injury by enhancing the mitochondrial dynamic imbalance, apoptosis, and inflammation, which favor subsequent damage [105]. The glucose level at admission is related to the severity of initial hemorrhage [106, 107]. Previous studies revealed that the initial hyperglycemia was an independent predictor of the occurrence of delayed cerebral ischemia (DCI) and poor outcome in SAH patients. The prognostic potential of the admission plasma glucose level was suggested to be beneficial in management protocols of SAH patients [108]. Insulin therapy improved the prognosis for patients with SAH. Antihyperglycemic treatment for keeping serum glucose in normal level may be worthwhile in patients with SAH, but more preclinical and clinical studies are needed to elucidate the role of hyperglycemia in SAH.

SAH is associated with disturbances in electrolyte and circulating blood volume homeostasis. Hyponatremia occurs in 10–34% of patients who experience SAH, which worsens their prognosis [109]. Such patients exhibit excessive natriuresis [110] and resultant osmotic diuresis, which leads to a decrease in systemic blood volume [111]. All patients with SAH demonstrated increased urine output and urinary excretion of sodium [26]. Adrenomedullin, a vasorelaxant peptide, is secreted into the CSF from the choroid plexus and can exert natriuretic effects in the kidney. CSF adrenomedullin concentration was significantly higher during the late period than during the early period following SAH [112]. Results demonstrated that late-period CSF adrenomedullin concentration correlated with hyponatremia and delayed ischemic neurological deficit by logistic regression analysis. After SAH onset, hyponatremia, but not a decreased circulating blood volume, was prevented by high sodium and water infusion, adapted to renal excretion. No significant correlations were found between hormone concentrations and natriuresis. The aim of the treatment of hyponatremia is maintenance of a positive salt balance and water replacement.

Low serum potassium levels were detected in approximately 50% of all SAH patients [66]. It is believed that hypokalemia results from the catecholamine surge after SAH. High level of circulating catecholamine leads to an excessive activation of the Na+/K+-ATPase via stimulation of *β*2-adrenergic receptor. The consequence is a shift of potassium ions from extracellular to intracellular spaces. Thus, a lower serum potassium concentration is found in SAH patients. The effect of potassium on outcome after SAH remains controversial. It was reported that the change of potassium level was not related to outcome or DCI after SAH. On the contrary, another study showed the relationship between serum potassium on outcome and DCI [113, 114]. Thus, in cases of severe hypokalemia, potassium should be supplemented either intravenously or orally.

In addition, hypomagnesemia at admission was associated with large amounts of extravasated blood volumes, longer duration of confusion, and poor clinical condition. A multivariate analysis revealed that hypomagnesemia at onset did not predict outcome; however, hypomagnesemia can predict DCI occurring between days 2 and 12 after SAH [115]. Patients with a high serum magnesium concentration had a reduced incidence of vasospasm as examined by angiography, but the difference did not reach statistical significance [116]. Magnesium is a neuroprotective agent for inhibiting vasospasm with the rationale that its vasodilatory action on vasospastic artery and improvement of cerebral blood flow result from the inhibition of calcium channels and of myosin light chain kinase [115]. However, a retrospective analysis observed that magnesium supplementation may not reduce the incidence of symptomatic cerebral vasospasm in patients with SAH [117]. Furthermore, the conclusion of a phase 3 randomized placebo-controlled and multicenter trial was not to recommend routine administration of magnesium, because intravenous administration of magnesium sulfate was not able to improve the overall clinical outcome after SAH [118].

### 3.4. Renal Dysfunction

Previously, renal dysfunction has been reported in 0.8% to 7% of SAH patients [10]. The one-year mortality was significantly higher in stroke patients with kidney damage than in those without kidney damage and increased along with the progression of renal insufficiency [119]. In addition, proteinuria is an independent predictor of one-year mortality rate in patients with stroke. In a retrospective analysis of a series of 787 SAH patients, a seemingly insignificant decrease in kidney function can adversely affect the 3-month outcome independently of other known predictors [120].

Renal failure was associated with volume loading and the aggressive maintenance of mean arterial pressure. In addition, SAH-induced sympathetic activation may play a crucial role in progression of renal failure [121, 122]. SAH patients frequently receive antibiotic therapy and undergo a significant number of contrast radiographic studies, including CT angiography, CT perfusion, and catheter-based digital subtraction angiography, which have been closely associated with renal dysfunction. The combination of these factors predisposes SAH patients to acute kidney injury. However, clazosentan, a potential drug for vasospasm after SAH, was found to be well tolerated by patients with severe renal impairment and in healthy subjects, which suggests no need for adjusting the dose of clazosentan in SAH patients even with severe renal damage [123].

Herein, we highlighted the importance of close surveillance of renal function and the value of renal hygiene in the SAH. We suggested renal protection strategy for SAH patients, including avoidance of redundant contrast-enhanced imaging examination, adequate hydration and renal protection, and caution usage of potentially nephrotoxic drugs and optimal dose of those with renal impairment.

### 3.5. Hematological Failure

In current literature, a high incidence of coagulative and fibrinolytic disorders was observed in patients with SAH, which was also associated with outcome. Several variables of coagulation and fibrinolysis were elevated after SAH. PT, APTT, and fibrinogen were in the normal range. A prospective study showed high level of plasmatic thrombin/antithrombin complex parallels clinical outcome [124]. Specifically, a generalized elevation of plasmatic D-dimer, an index of subarachnoid clot lysis, was invariably found. Hence, D-dimer was a useful laboratory tool for assessing clinical status, since it was correlated with patients' long-term outcomes [125].

## 4. Prospective and Conclusion

With improvements of neurocritical care in SAH, we recommend that more attention should be shifted to nonneurological complications. First, animal model of SAH that mimics the pathophysiology after SAH will be an invaluable tool. The limitations of recent models must be carefully considered. First, the nature of aneurysm rupture is sudden and unpredictable; however, there are no naturally occurring animal models of SAH. Generally, SAH animal models used two major techniques to simulate SAH: an injection model and a vascular perforation model. The injection model neglects the importance of the injury to the artery in the pathophysiology of SAH, has high risk of mechanical damage to brain tissues, and requires craniotomy. The drawbacks of endovascular puncture model are large variations in the severity of bleeding and a high mortality rate. Besides, Wada et al. established a mouse model of intracranial aneurysm that the rupture of aneurysms would occur within a predictable time course [126]; nevertheless, it requires more experiments. Secondly, various species of SAH models are different in genome, anatomy, and physiology from humans. In addition, young animals without other diseases are used, but SAH patients often present with other diseases, such as hypertension, diabetes, and cardiopathy. At last, each model has its priority to study certain aspects of the pathophysiological process behind SAH. Future studies should differentiate suitable SAH models that target nonneurological complications. Secondly, the mechanisms of those nonneurological complications after SAH need further study. Although sudden increase in cardiac sympathetic nervous activation was believed to be the most important mechanism, it is seemingly difficult to measure it in humans [127]. It only relies on indirect method by measuring the level of circulating catecholamines. Further studies are needed to explore this issue in SAH patients. Thirdly, most current results come from retrospective analyses, which have many methodological shortcomings of purely retrospective studies. Thus, it is extremely important to execute large, double-blind, randomized, prospective trials evaluating the frequency, severity, role, and therapeutic strategy of nonneurological medical complications after the rupture of aneurysms [128, 129]. Furthermore, severity of illness scores is frequently used for daily assessment in SAH patients, such as Sequential Organ Failure Assessment (SOFA) score and APACHE II score [130, 131]. The efficiency of those scores requires validation in SAH populations. Recently, for patients with SAH, treatments commonly involve the management of intracranial hypertension and the support of cerebral perfusion pressure with volume loading and inotropes. However, cerebral perfusion pressure-targeted management of intracranial hypertension in SAH patients may lead to nonneurological complication (e.g., NPE) and eventually worsen outcome [132]. Thus, we stressed that potential adverse effects of currently management strategy may offset their beneficial effects. We suggest developing a more efficient treatment strategy before it is too late. At last, we emphasized that the physician should keep the nonneurological complication after SAH in mind. For example, if a patient was diagnosed as having acute myocardial infarction with ECG changes and troponin elevation, who also presented neurologic symptoms or signs, brain computed tomography should be performed to exclude SAH before the thrombolytic therapy.

## 5. Conclusion

SAH is not only affecting brain tissue, but also impairing extracerebral organs. Extracerebral complications are associated with the high mortality rates and neurological impairments following SAH, even after adjustment for the severity of the initial neurological injury. All of the nonneurologic complications have been linked to adverse clinical outcomes, such as circulatory failure, NPE, electrolyte imbalance, or hyperglycemia.
